# The role of silicon in regulating physiological and biochemical mechanisms of contrasting bread wheat cultivars under terminal drought and heat stress environments

**DOI:** 10.3389/fpls.2022.955490

**Published:** 2022-08-03

**Authors:** Waseem Ashfaq, Sigfredo Fuentes, Graham Brodie, Dorin Gupta

**Affiliations:** School of Agriculture and Food, Faculty of Veterinary and Agricultural Sciences, The University of Melbourne, Parkville, VIC, Australia

**Keywords:** *Triticum aestivum*, silicon, oxidative stress, stress alleviation, stress adaptation, abiotic stress, antioxidants

## Abstract

The individual and cumulative effects of drought stress (DS) and heat stress (HS) are the primary cause of grain yield (GY) reduction in a rainfed agricultural system. Crop failures due to DS and HS are predicted to increase in the coming years due to increasingly severe weather events. Plant available silicon (Si, H_4_SiO_4_) has been widely reported for its beneficial effects on plant development, productivity, and attenuating physiological and biochemical impairments caused by various abiotic stresses. The current study investigated the impact of pre-sowing Si treatment on six contrasting wheat cultivars (four drought and heat stress-tolerant and two drought and heat stress-susceptible) under individual and combined effects of drought and heat stress at an early grain-filling stage. DS, HS, and drought-heat combined stress (DHS) significantly (*p* < 0.05) altered morpho-physiological and biochemical attributes in susceptible and tolerant wheat cultivars. However, results showed that Si treatment significantly improved various stress-affected morpho-physiological and biochemical traits, including GY (>40%) and yield components. Si treatment significantly (*p* < 0.001) increased the reactive oxygen species (ROS) scavenging antioxidant activities at the cellular level, which is linked with higher abiotic stress tolerance in wheat. With Si treatment, osmolytes concentration increased significantly by >50% in tolerant and susceptible wheat cultivars. Similarly, computational water stress indices (canopy temperature, crop water stress index, and canopy temperature depression) also improved with Si treatment under DS, HS, and DHS in susceptible and tolerant wheat cultivars. The study concludes that Si treatment has the potential to mitigate the detrimental effects of individual and combined stress of DS, HS, and DHS at an early grain-filling stage in susceptible and tolerant wheat cultivars in a controlled environment. These findings also provide a foundation for future research to investigate Si-induced tolerance mechanisms in susceptible and tolerant wheat cultivars at the molecular level.

## Introduction

Bread wheat is an important staple food crop in most parts of the world. With a total grain production of around 760 million tons in 2020 (FAOSTAT, 2020), wheat grain provides ~20% of the total vegetal calories and proteins for nearly 4.5 billion people worldwide (Shiferaw et al., 2013). However, global wheat productivity faces challenges due to climatic adversities, which are becoming more acute in most of the world's established agricultural regions, raising concerns for future food security (FAO, 2019). In a rainfed cropping system, temperature and water availability play a critical role in determining optimum crop growth and productivity (Rezaei et al., 2015; Tricker et al., 2018).

Abiotic stress factors, such as drought and heat are the primary cause of grain yield (GY) reduction worldwide, and the frequency of their concomitant effects has increased in the semi-arid wheat belts of the world (Fahad et al., 2017). Due to the expected rise in global temperature (Buis, 2019; NOAA, 2020; IPCC, 2022) and change in the precipitation patterns of dryland agriculture (Rojas et al., 2019), the individual and combined effects of drought and heat stresses would disrupt morphological, biochemical, and physiological processes more than any other environmental stresses (Zandalinas et al., 2018; Sattar et al., 2020). Drought and heat stress-induced altered/affected processes during grain filling includes a reduction in photosynthesis, various oxidative damages to cell organelles (mitochondria, chloroplast), earlier senescence, shortening of the plant life cycle, and significant losses in the grain yield (Barnabás et al., 2008; Rezaei et al., 2015; Tricker et al., 2018). The exposure of plants to drought stress (DS) and heat stress (HS) disturbs the reactive oxygen species (ROS) quenching ability of different antioxidants, leading to excessive production of several ROS. As a result of this overproduction, ROS intracellular levels rapidly increase and negatively affect various cellular metabolic processes (Sharma et al., 2012; Alzahrani et al., 2018). Plants with increased antioxidants, either in constitutive or induced forms, have demonstrated maximum tolerance against oxidative stress damages to cells and organelles. Higher antioxidants level also supports the accumulation of various osmolytes, such as amino acids, soluble sugars, proline, and different organic acids (Biju et al., 2017).

Plant responses to combined drought and heat stress (DHS) are very complicated, and the intensity of one stress might alter responses for the other. Combined stress has significantly higher detrimental and negative impacts on plant phenology and physiology. The combined effects of DHS resulted in suppressing various physiological processes, i.e., net photosynthetic rate and higher canopy temperature (T_c_), which causes disproportionate plant development and productivity compared to individual stress effects (Barnabás et al., 2008; Prasad et al., 2008; Zandalinas et al., 2018; Sattar et al., 2020). Various antioxidants and osmolytes induced a tolerance response in the plants against individual stresses; however, the functioning of these response mechanisms was altered significantly under combined stress. Higher yield penalties have been linked with an extended period of heatwaves over 32°C, coinciding with DS during anthesis and grain-filling stages (Perdomo et al., 2015; Tricker et al., 2018).

Silicon (Si) is the second most ubiquitous element (31%) in the geosphere after oxygen (49%), and it exists mainly in the form of silicates (Luyckx et al., 2017). Although Si has not been recognized as an essential plant element, the benefits of Si application in improving the development and yield of many plant species are well-documented (Cooke and Leishman, 2016; Debona et al., 2017; Luyckx et al., 2017). Plant roots can uptake Si in uncharged mono silicic acid form (H_4_SiO_4_) *via* aquaporin channels (NIPs: Nodulin26-like intrinsic proteins) when the pH of the growth medium is below 9. The NIPs, which belong to the major intrinsic proteins, form highly selective transport channels to facilitate the passive transport of moisture and uncharged molecules (Grégoire et al., 2012; Deshmukh et al., 2013), including silicic acid (Ma et al., 2006). Mono silicic acid is a weak acid in an aqueous solution, mainly accumulated in cell walls and plant tissues. However, Si concentration in plants varies significantly from 0.1 to 10% of the total shoot dry weight (DW) (Hodson et al., 2005).

Several research studies showed that plants with a high Si ratio in the roots and shoots are less prone to abiotic stresses (Debona et al., 2017; Etesami and Jeong, 2018). The Si accumulation in plants can alleviate the harmful effects of various abiotic stresses by targeting oxidative stress reduction through enzymatic regulations (Biju et al., 2017), enhanced antioxidant activities (Biju et al., 2017; Etesami and Jeong, 2018), modification of gas exchange apparatus (Cooke and Leishman, 2016; Debona et al., 2017), various osmotic adjustments, improved nutrient uptake, and regulation of different osmolytes (Rizwan et al., 2015; Cooke and Leishman, 2016; Etesami and Jeong, 2018). A meta-analysis of 145 research studies concluded that Si application significantly improved DW, chlorophyll biosynthesis, net assimilation rate, and antioxidants activity in several plant species grown under various abiotic stresses (Cooke and Leishman, 2016). Si application also proves to have beneficial effects in maintaining plant water status (Etesami and Jeong, 2018), computational water stress indices (Ashfaq et al. unpublished data), GY, and yield-related components under abiotic stresses (Cooke and Leishman, 2016).

Although the response of the wheat crop to individual DS and HS has been quite comprehensively investigated in recent years, the combined effects of these stresses on wheat morphology and physiology have received limited attention (Mahrookashani et al., 2017; Urban et al., 2018). The ability of Si to enhance drought tolerance by regulating the physiological and biochemical mechanisms is known in many crops, including monocots (rice, wheat) and dicots (lentils, soybean) (Debona et al., 2017). First, the Si role against the combined effects of drought and heat in wheat has not been studied to date (Frew et al., 2018). Second, complex mechanisms and their interactions in Si-mediated tolerance against the individual and the combined effects of DS and HS in wheat need to be addressed at the physiological and biochemical levels at critical growth stages to estimate the impact on GY correctly. The study aimed to investigate the Si-mediated tolerance in wheat against individual and combined effects of terminal DS and HS at physiological and biochemical levels in a controlled environment.

## Materials and methods

### Plant materials

Six contrasting wheat cultivars, RAC875, Excalibur, ECH957, RAC622, Kukri, and CM59443, were used as experimental materials. RAC875 and Excalibur have shown relatively stable yield performance under the drought and heat-adapted environment of South Australia (SA). At the same time, Kukri, a hard wheat cultivar, suffers significant GY losses under moderate to low rainfall environments of SA (Fleury et al., 2010). RAC875, Excalibur, and Kukri show similar phenology (3–5 days heading time difference) under optimum growing conditions and possess RHt2 semi-dwarfing genes. These South Australian wheat cultivars were also comprehensively characterized and physiologically investigated by Izanloo et al. (2008). Similarly, cultivars, i.e., ECH957 and RAC622, showed relatively stable morpho-physiological performance and GY in controlled environment DS and HS experiments. Conversely, CM59443 endures significantly lower yield performance in similar DS- and HS-controlled environment experiments (Ashfaq et al., unpublished data).

### Experimental design and establishment

To determine the role of Si in response to terminal DS, HS, and DHS on contrasting wheat cultivars, an experiment was conducted in a controlled greenhouse at Dookie Campus, the University of Melbourne (−36.384 S, 145.707 E) from July to November in 2019. A factorial experimental arrangement in a complete randomized design was used with three replications. Before sowing, 4 mM of Si solution was prepared using distilled water (dH_2_O) at pH 7.0 (Deshmukh et al., 2013) and applied at two levels, i.e., Si_0_ = 0 mM and Si_1_ = 4 mM (Alzahrani et al., 2018). Respective Si solutions were thoroughly mixed in the individual respective treatment pot growth medium (standard potting mix, pH ~ 6.0) using a spray bottle.

Five uniformly selected seeds were sown in each polypropylene square-shape perforated nursery pot (466 cm^3^ volume), containing the same mass (2 kg) of the oven-dried standard potting mix. Polylining was applied to the bottom of the pots to prevent Si from leaching. Appropriate fertilization (ICL Peters Excel CalMag grower water-soluble fertilizer) was applied before sowing, followed by three doses at tillering, jointing, and booting stage to ensure recommended nutrition supply for optimum plant growth.

To estimate pot field capacity, two perforated pots filled with 2 kg of the oven-dried potting mix were overflowed with tap water and allowed to drain for 12 h. When the water stopped dripping, the potting mix from both pots was weighed separately for their saturated weight and oven-dried (70°C for 36 h) for their DW. The percent moisture contents of the potting mix were measured using Equation 1 as described by Topp et al. (2008).


(1) $$\begin{array}{l} \text{Soil water content }\left(\%\right)=\;\\ \frac{\left[\text{potting mix saturated weight }(\text{g})\text{ -potting mix dry weight }(\text{g}\right)}{\text{potting mix dry weight}(\text{g})]}\times 100 \end{array}$$

The day- and night-time temperatures in a glasshouse were maintained at 24 ± 1 and 18 ± 1°C (13/11), respectively, to replicate the average Australian wheat belt growing environment for optimum plant growth. All pots were watered up to full-field capacity daily until the Zadok's scale growth stage 75 (10 days after complete anthesis of the main tiller) (Zadok stage 75: Zadoks et al., 1974).

Plants were monitored individually to determine when their main tiller reached 10 days after complete anthesis (Zadok stage 75: Zadoks et al., 1974; Telfer et al., 2015); as in the Mediterranean environments, the wheat yield is determined mainly by the main stem contribution. At this stage, single extreme events, such as DS, HS, and a combination of both, DHS were imposed on the designated pots of the experiment. For DS treatment, drought with 38 ± 3% field capacity was imposed on the DS, and DHS selected pots for 14 days. Before DS treatment, irrigation was gradually reduced for 5–7 days for acclimation to attain 38 ± 3% field capacity. Pots were weighed daily to measure the mass of moisture loss through evapotranspiration. Based on moisture loss, pots were watered accordingly to maintain the required field capacity. The effects of Si and DS on the tolerant and susceptible wheat cultivars were monitored by destructive (estimation of relative water content; RWC) and non-destructive sampling (measure of chlorophyll fluorescence and flag leaf senescence with Mini-Palm-II and portable SPAD-502, respectively).

For HS treatment, designated pots of HS and DHS group were shifted to separate plant growth chambers (Conviron, PGR15) 1 week before HS treatment to acclimatize with the PGR15 growing environment. Plant growth chambers were programed at 24 ± 1°C and 18 ± 1°C (13/11 h) for light and dark periods to support circadian clock genes expressions (Izanloo et al., 2008). The PGR15 was equipped with high-pressure sodium lamps for red, blue, and far-red light spectrum composition and high-power discharge metal halide. Light intensity was adjusted to 500–550 μ mol/m^2^/s at the top of the plant canopy to supplement natural radiation (Watson et al., 2018). The RH of PGR15 was set according to the glasshouse. Both HS and DHS group plants were subjected to high-temperature acclimation for 3 days with day/night temperatures of 36/22°C for 8 and 12 h, respectively, to mimic the hot day events which are common in the SA wheat belt (Spiertz et al., 2006; Telfer et al., 2015). Before and after 8 h of HS of 36°C, growth chambers were programed in a cyclic pattern of 28 and 32°C for 1 h each for a gradual increase and decrease in temperature. The RH was set at 45–50% during HS. In the DHS group, DS with 38 ± 3% field capacity was also maintained along with HS for the combined stress treatment. At the same time, irrigation was applied to the HS group to remove any confounding effect of drought. After 14 days of DS, normal irrigation was resumed in the DS and DHS groups. After the HS treatment, pots were shifted back to the glasshouse for the remaining growth cycle. During the imposed period of DS, HS, and DHS, water was normally applied to control (C) pots with full-field capacity. During the experiment, the pots were shuffled manually inside the glasshouse after every 2 weeks to avoid pseudo replications and border effects.

### Plant sampling

On the last day of DS, HS, and DHS, the sampled leaves were taken from each replicate and stored at −80°C for the assay of enzymatic antioxidants, total soluble sugars (TSS), fructose contents (FCs), free amino acid (AA), and proline concentration.

### Chlorophyll content, chlorophyll fluorescence, and relative water content measurements

Except for RWC, measurements were made non-destructively using flag leaves from each replicate on the last day of the stress treatments. The chlorophyll content of flag leaves was estimated using a SPAD-502 Plus (Konica Minolta, Japan). Chlorophyll content (SPAD value) was measured in intact expanded flag leaves at different leaf positions. A rapid, non-invasive chlorophyll fluorescence assessment was done using a portable red LED modulation fluorescence system (Mini-Palm-II with leaf clip holder, Heinz Walz, Germany). Chlorophyll fluorescence was determined (means of five measurements) 1 day before the end of the stress treatments. Minimal chlorophyll fluorescence (F_o_) for 30 min of dark-adapted leaves was measured by using a pulse of low-intensity light (>0.1 μmolm^−2^ s^−1^). After that, maximal chlorophyll fluorescence (F_m_) was recorded by applying a pulse of saturated white light (>3,000 μmolm^−2^ s^−1^) for 1 s in the disks of the same leaves. The quantum yield (F_v_/F_m_) of PSII for dark-adapted leaves was estimated as described by Carvalho and Amâncio (2002).


(2) $$\text{Fv/Fm}=\frac{\left({\text{F}}_{\text{m}}-{\text{F}}_{\text{o}}\right)}{{\text{F}}_{\text{m}}}$$

Where F_o_ corresponds to the minimal fluorescence level, F_m_ is the maximum possible level of fluorescence, and F_v_ is the variable fluorescence under stress.

The RWC was estimated by following the standard method described by Barrs and Weatherley (1962). Two samples (8–10 cm long) were taken from a mid-section of developed leaves. Fresh weight (FW) was determined instantly after excision, followed by placing in falcon tubes containing 10 ml of water (H_2_O) for 12 h in a standard room environment. Following hydration, the samples were taken out from the H_2_O, gently dried with tissue, and weighed immediately for turgid weight (TW). Then, the samples were oven-dried at 70°C for 72 h to determine their DW. The RWC was calculated using Equation 3 as below.


(3) RWC (%)  =  [(FW-DW)/(TW-DW)]  ×  100

### Computational water stress indices through infrared thermography

**Figure 1:**
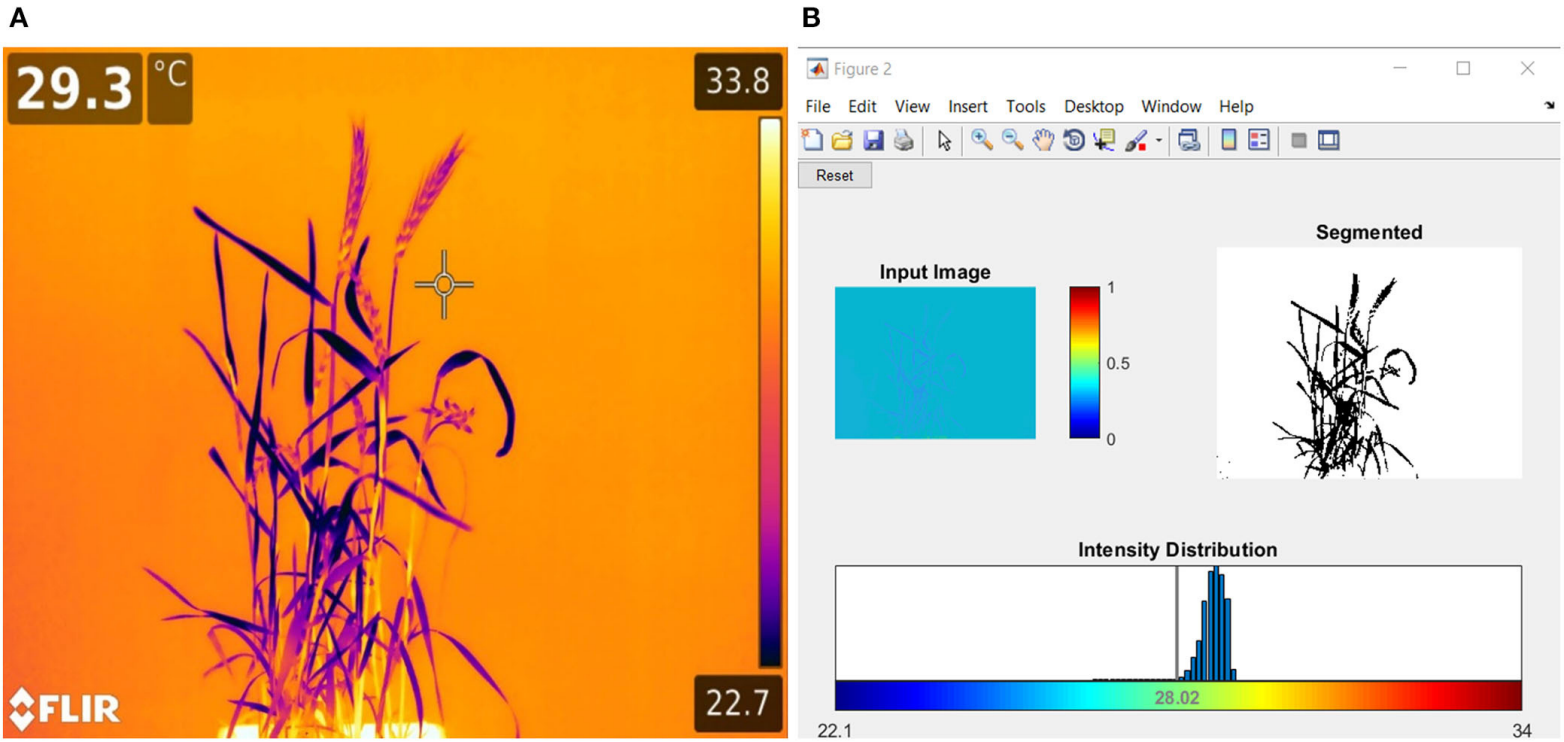
**(A)** Sample thermal image and **(B)** image analysis graphic user interphase (GUI) used for crop water stress index (CWSI), canopy temperature (T_c_), and canopy temperature depression (CTD) calculations of plants through a customized code written in MATLAB (Fuentes et al., 2012).

On the last day of DS, HS, and DHS, infrared thermal imaging (IRTI) of each replicate was taken by using a high-performance handheld thermal imaging camera (T-series, Model 1050sc, with true HD 1,024 ×768 pixels resolution) (FLIR Systems AB, Täby Sweden) (FLIR, 2015). The thermal sensitivity (NETD) of the 1050sc camera was <20 mK at 30°C with a 0.47 milliradians spatial resolution (IFOV). The object temperature measurement range was between −40 and 150°C. Each pixel of the image holds thermal reading in units of °C. All IRTI were taken from a constant distance of 2 m to view the plant at a 30° angle from the horizontal direction. All thermal images were taken in standard room conditions to avoid plant canopy and leaf temperature acclimatization with the surrounding environment and minimize pixel temperature variations. Each thermal image was then converted to .dat files. Data were extracted using MATLAB code scripted in MATLAB R2017b (Math works Inc. Natick, Massachusetts, USA) through the image analysis toolbox (Figure 1). While analyzing the images for canopy temperature (T_c_), canopy temperature depression (CTD), and crop water stress index (CWSI), the background temperature effects were subtracted by setting the minimal and maximal thermal pixels within the canopy in the thermal histogram. The CWSI was calculated by following the protocols established by Fuentes et al. (2012).


(4) $$\text{CWSI }=\;\frac{\left(\text{Tc-Tw}\right)}{\left(\text{Td-Tw}\right)}$$

Where T_c_ is the canopy temperature of each replicate, T_w_ and T_d_ are the corresponding temperature (°C) of the control and stressed plants, respectively.

While CTD was calculated by subtracting the T_c_ from the mean environmental temperature (T_a_), it is positive when T_c_ is lower than the surrounding environment.


(5) CTD=Ta-Tc

### Determination of TSS and FC

The TSS and FC were determined spectrophotometrically at 620 and 420 nm, respectively, by following the protocol proposed by Dubois et al. (1951). Frozen leaf samples (0.2 gm) were taken and homogenized with TissueLyser II (Qiagen, Germany) in 4 ml of 80% (v/v) ethanol (C_2_H_5_OH). The extract was centrifuged at 4,000 rpm for 20 min, and the supernatant was pooled in a flask by repeating the procedure thrice. The total volume of the flask with 80% ethanol was maintained at 10 ml. The TSS and FC were determined using the anthrone reagent and H_2_SO_4_, respectively, followed by incubation at 95°C for 15 min. Absorbance readings were recorded using a single beam UV scanning spectrophotometer (Halo RB-10, Dynamica Scientific Ltd.). The concentration of TSS and FC was calculated from the standard curve and expressed as mg g^−1^ of FW.

### Determination of proline and free AA

The free level of proline and AA were determined spectrophotometrically at 520 and 570 nm, respectively, by following the method of Bates et al. (1973). Frozen leaf samples (0.2 gm) were homogenized with TissueLyser-II (Qiagen, Germany) in 5 ml of 3% aqueous sulfosalicylic acid (SA) solution, followed by centrifugation at 4,000 rpm for 15 min. An aliquot of 2 ml was reacted with a 4 ml solution (2 ml each) of glacial acetic acid and acid ninhydrin in a 20 ml glass tube at 100°C. After 1 h, the reaction was terminated in an ice bath. After that, 4 ml of toluene was added to the reaction mixture (RM) and mixed vigorously with a glass tube stirrer for 30 s. After 1 h, when toluene was aspirated, absorbance was recorded using a single beam UV spectrophotometer (Halo RB-10, Dynamica Scientific Ltd.). The blank was prepared by adding toluene in place of sample extract. The standard curves for proline and amino acids were prepared by dissolving different concentrations (10–50 μg/ml) of proline and leucine in 3% of SA, respectively. Proline extracts and AA concentrations were estimated from the standard curve and calculated on an FW basis.


(6) $$\begin{array}{l} \mu \text{mol proline/g FW sample = }\\ \;\frac{\left[\left(\mu \text{g proline/ml}\times \text{toluene }(\text{ml})\right)\text{/115}.\text{5 }\mu \text{g/}\mu \text{mol}\right]}{\left[\left(\text{g sample}\right)\text{/5}\right]} \end{array}$$

### Assays for antioxidant enzymatic activities

The peroxidase (POX) activity was estimated by measuring the mmol guaiacol (C_7_H_8_O_2_) mg^−1^ protein min^−1^. Enzyme extract (200 μl) was added to an RM containing 2 ml of 0.1M Sodium phosphate buffer (Na-PB) (pH 6.8), 50 μml of 10 mM H_2_O_2_, and 0.95 ml of 20 mM guaiacol. POX activity was assayed spectrophotometrically at 470 nm by measuring the change in the absorption for 3 min due to an increase in the oxidation of C_7_H_8_O_2_ in the presence of H_2_O_2_ (Chance and Maehly, 1955). For ascorbate peroxidase (APX) activity estimation, enzyme extract (250 μl) was added to an RM of 0.05 M of Na-PB (pH 7.2), 30% H_2_O_2_ (v/v), and 0.5 mM ascorbic acid (ASC). Ascorbate peroxidase activity was estimated in mmol ascorbate mg^−1^ protein min^−1^ by quantifying the ASC oxidation with a reduction in absorbance for 2 min difference at 290 nm (Chen and Asada, 1989). Superoxide dismutase (SOD) activity was assayed spectrophotometrically at 560 nm by following Dhindsa et al. (1981). The enzyme extract (300 μl) was added into an RM of 0.3 ml of 130 μM of methionine, 1.5 ml of 50 mM of Na-PB (pH 7.8), 0.3 ml of 100 μM of methylene diamine tetraacetic acid, 0.3 ml of 20 μM riboflavin, 0.3 ml of 750 μM of nitro-blue tetrazolium (NBT), and 100 μl of dH_2_O. A 20-watt fluorescent lamp then illuminated on the reaction tubes for 20 min. The reaction was terminated by incubating tubes in dark conditions by covering them with aluminum foil. One unit of SOD (mg^−1^ protein min^−1^) is equal to the enzyme amount involved in 50% inhibition of the NBT reduction compared to the control. The catalase (CAT) activity was assayed by adding 250 μl of enzyme extract to the RM having 1.5 ml of 50 mM of Na-PB (pH 7.8), 300 μl of 0.1 M H_2_O_2_, and 1 mL of dH_2_O. The change in the absorbance in 2 min at 240 nm was recorded as a measure of CAT activity (Chance and Maehly, 1955).

### Determination of grain yield and yield components

At maturity, all five plants from each pot were cut at the surface level and weighed to record above-ground biomass. Grain yield (GY; gm pot^−1^) was measured as the weight of harvested grains from all five plants within each replicate. Thousand grains weight (TGW, gm), grain number per spike (GNS), three spikes grains weight (GWS, gm), and the number of spikelets per spike (NSPS) (three spikes average) were also determined. Harvest index (HI) was recorded as GY divided by the total above-ground dry matter (Yue et al., 2006).


(7) $$\text{HI }\left(\%\right)\text{ = }\frac{\text{ Grain yield }}{\text{Total above ground biomass}}\text{ ​​ }\times \text{ 100}$$

### Statistical analysis

Statistical significance of the variation of the trait with repeated measures was tested by factorial analysis of variance (ANOVA) for treatment effects (*p* < 0.05) by keeping Si and stress treatments as fixed factors within the cultivars. A least significant difference (LSD) and pairwise comparison test were used for comparing treatment means, standard error (SE) of the means, and their interaction effect calculated through “agricolae” package of R statistical software (Mendiburu, 2020; R Core Team, 2020). Thermal image data were derived using MATLAB code scripted in MATLAB R2017b (Mathworks, 2017) through Images Analysis Toolbox for T_c_, CTD, and CWSI (Fuentes et al., 2012) and analyzed through R software. The “ggplot2” package was used for plotting and data visualization (Wickham, 2016).

## Results

### Effect of Si on chlorophyll fluorescence, chlorophyll content, and RWC

**Figure 2:**
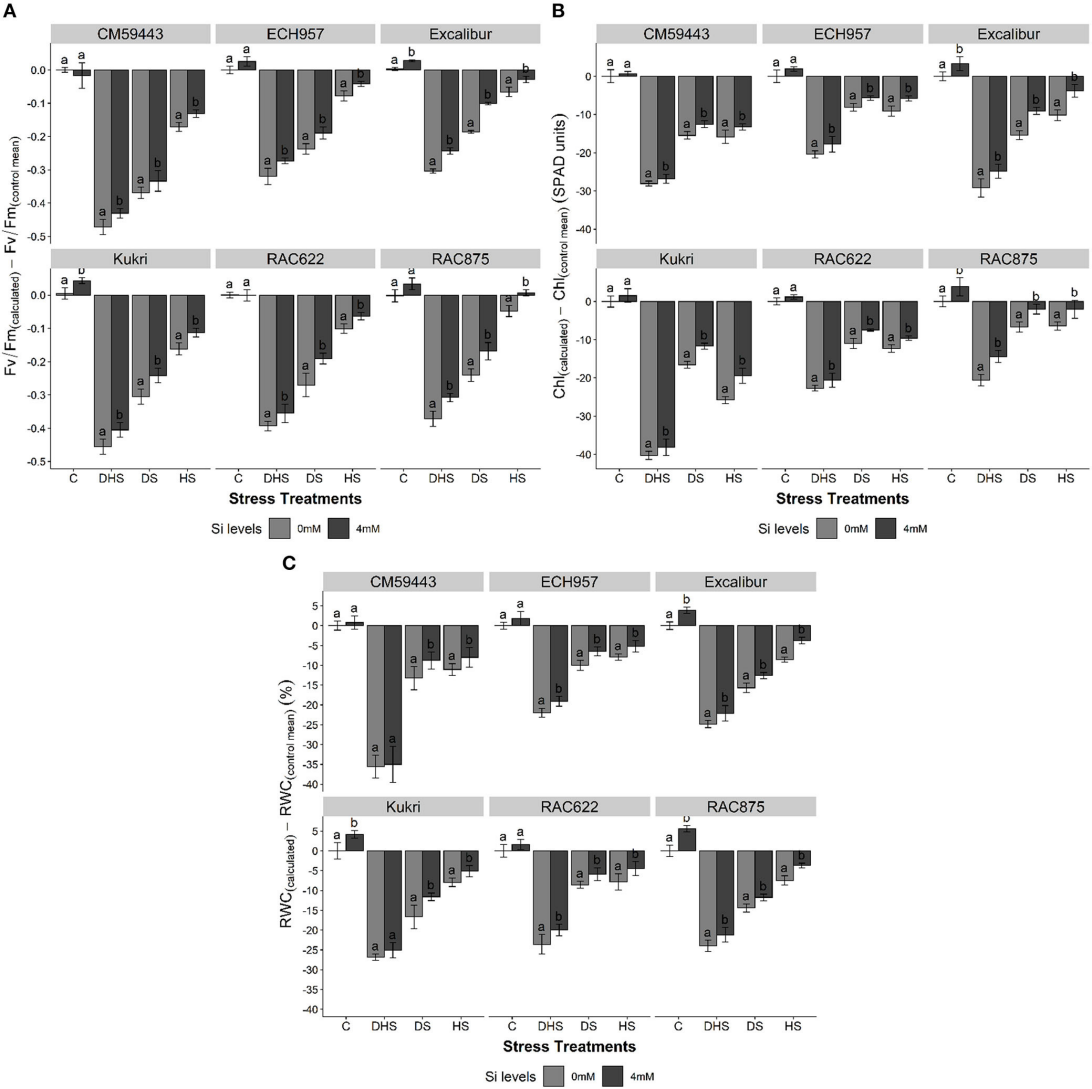
**(A–C)** Effect of silicon on **(A)** chlorophyll fluorescence (F_v_/F_m_), **(B)** chlorophyll content (SPAD unit), and **(C)** relative water content (RWC) (%) of six contrasting wheat cultivars under terminal drought stress (DS), heat stress (HS), and drought-heat combined stress (DHS) in a controlled glasshouse condition. F_v_/F_m_ is a mean of *n* = 9, SPAD value is a mean of *n* = 27. Mean values are presented with ± standard error (SE); SE represents error bars. Different letters indicate significant differences within the cultivars at *p* < 0.05.

Overall, Si treatment recovered the efficiency of PSII with a difference in the extent and ability of a cultivar under experimental stress treatments. The quantum yield of the flag leaves of the cultivars showed a net drop with the onset of the stress treatments. However, Si treatment significantly (*p* < 0.001) enhanced the F_v_/F_m_ under DS, HS, and DHS. Under DSSi, a maximum increase, compared to susceptible cultivars (Kukri, 12.8%; CM59443, 7.9%), was observed in RAC622 (14.3%) and Excalibur (14.1%). Interestingly, the percent increase under HSSi was almost the same across the tolerant and susceptible cultivars (6.5%). However, under DHSSi, a maximum increase was observed in the susceptible cultivar, Kukri (14.8%), followed by tolerant cultivars (RAC875, 14.3; Excalibur, 12.4%; ECH957, 9.6%, and RAC622, 8.8%) (Figure 2A).

A declining behavior in chlorophyll content (SPAD readings) was observed throughout the stress treatment. Under C, all cultivars, except RAC875 and Excalibur, showed a non-significant increase with Si treatment. Compared with C, the highest decrease in the chlorophyll content was observed under DHS, followed by HS and DS across the cultivars. However, Si treatment significantly (*p* < 0.001) increased the chlorophyll content across the cultivars regardless of the stress treatments. With Si treatment, the highest chlorophyll content percent increase was observed in Kukri (DSSi, 11.1%; HSSi, 18.3%, and DHSSi, 10.8%) followed by RAC875 (DSSi, 7.8%; HSSi, 7.2%, and DHSSi, 13.4%) and Excalibur (DSSi, 11.6%; HSSi, 10.7%, and DHSSi, 10.4%) (Figure 2B).

Under C, the RWC among studied cultivars ranged between 80.5 and 86.9%. However, RWC progressively declined under DS, HS, and DHS compared to C (Figure 2C). The decreasing trend was more evident among susceptible cultivars (CM59443, 79.1% and Kukri, 47.0%) compared to the tolerant cultivars (Excalibur, 40.0%; RAC622, 39.3%; RAC875, 38.6%; ECH957, 36.2%). The Si treatment significantly improved RWC under HS compared to C treatment, followed by DS and DHS and the results were more pronounced in susceptible cultivars compared to tolerant cultivars. Under DS, Kukri and CM59443 had 7.5 and 6.6% increase in RWC, respectively, than ECH957 (4.9%), Excalibur (4.3%), RAC875 (3.7%), and RAC622 (3.6%). Similarly, under HS, the maximum percent increase was observed in Excalibur (6.2%), followed by RAC875 (4.8%) and RAC622 (4.5%). However, under DHSSi, only tolerant cultivars showed a significant increase in RWC (RAC622, 6.0%; ECH957, 4.8%; RAC875, 4.5% and Excalibur, 4.4%), and susceptible showed a non-significant increase in RWC.

### Effect of Si on computational water stress indices

**Figure 3:**
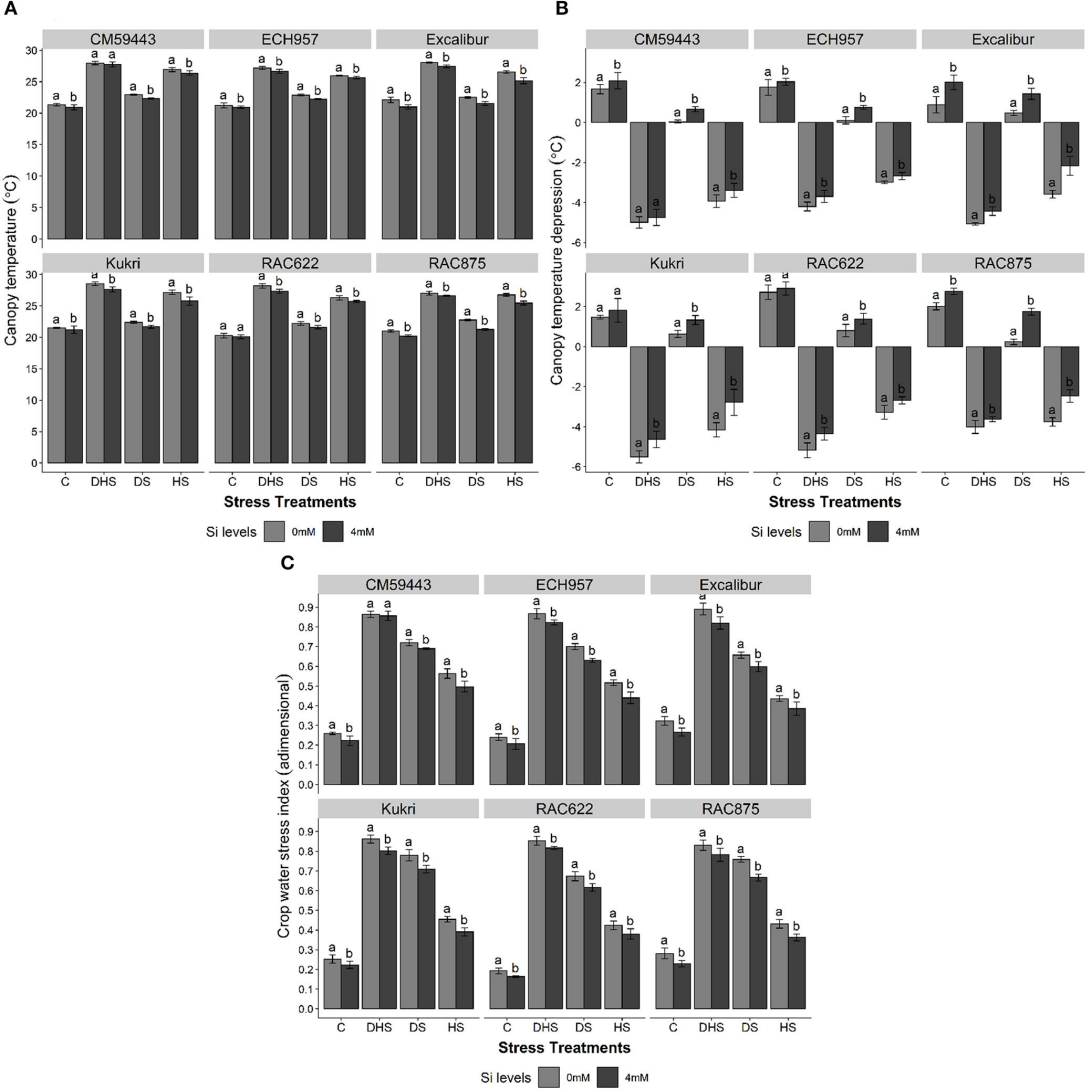
Effect of silicon on **(A)** plant's canopy temperature (°C), **(B)** canopy temperature depression (°C), and **(C)** crop water stress index (adimensional) of six contrasting wheat cultivars under terminal drought, heat and drought-heat combined stress in controlled glasshouse conditions. Mean values are presented with ± SE, represented with error bars. Different letters indicate statistical significance within each cultivar at *p* < 0.05.

The IRTI analysis outputs (T_c_, CTD, CWSI) are shown in Figures 3A–C. Cultivars exhibited significant differences (*p* < 0.001) for T_c_ with Si under all stress treatments. In a studied panel, the values of T_c_ under C ranged from 20.28 to 22.11°C. Under DHS, higher T_c_ values were recorded in susceptible cultivars (CM59443, 29.0°C and Kukri, 28.5°C), followed by RAC875 and Excalibur (28.1°C). Under DS and HS, T_c_ values of susceptible cultivars ranged from 22.4 to 23.0°C and 26.9–27.2°C, respectively, compared with 22.2–22.9°C and 26.3–26.8°C among tolerant cultivars, respectively. However, a significant reduction in T_c_ was observed with Si treatment under all stress treatments. On average, under all stress treatments, Excalibur performed better with Si (4.4% reduction in T_c_), followed by RAC875 (4.3% reduction in T_c_) and Kukri (3.3% reduction in T_c_). Except for CM59443 under DHS, both susceptible cultivars significantly reduced T_c_ across the stress treatments after Si treatment (Figure 3A).

Similarly, all cultivars showed lower CTD values under DS, HS, and DHS. However, Si treatment showed a positive effect in improving the CTD across the stress treatments (Figure 3B). The positive CTD values were observed under C and DS, whereas HS and DHS showed negative CTD values across the cultivars. Under HS, the tolerant cultivars exhibited almost the same CTD levels (~-3.5°C) compared with ~-4.0°C in the susceptible cultivars. However, a significant improvement (*p* < 0.001) in CTD was observed with Si treatment under DS, HS, and DHS across the cultivars. The maximum increase with Si treatment under DSSi was observed in RAC875 (62.3%), followed by 39.5% under HSSi in Excalibur and 16.0% under DHSSi in RAC622. A similar increasing trend was observed in susceptible cultivars, except for a non-significant increase in CM59443 under DHSSi.

A significant effect (*p* < 0.001) of Si was observed among stress treatments and their respective control in improving CWSI (Figure 3C). The highest CWSI values were observed under DHS and ranged between 0.83 and 0.89, respectively, followed by 0.65–0.77 and 0.42–0.56 under DS and HS, respectively. However, results showed that Si treatment significantly normalized the CWSI across the stress treatments. Under HSSi, the maximum reduction among tolerant cultivars was observed in RAC875 (16.0%), followed by ECH957 (14.8%), Excalibur (11.7%), and RAC622 (10.2%). The susceptible cultivars, Kukri and CM59443, also showed a significant decrease under HSSi in CWSI by 14.1 and 11.8%, respectively. Similarly, under DSSi, the same decreasing trend in CWSI was observed for all tolerant and susceptible cultivars. Among tolerant cultivars, the maximum reduction in CWSI was observed in RAC875 (12.1%) compared with the susceptible cultivar Kukri (9.0%). Under DHSSi, Excalibur, among the tolerant cultivars, and Kukri, among the susceptible cultivars, performed better with an overall decrease of 7.9% and 7.0%, respectively.

### Effect of Si on osmolytes accumulation

**Figure 4:**
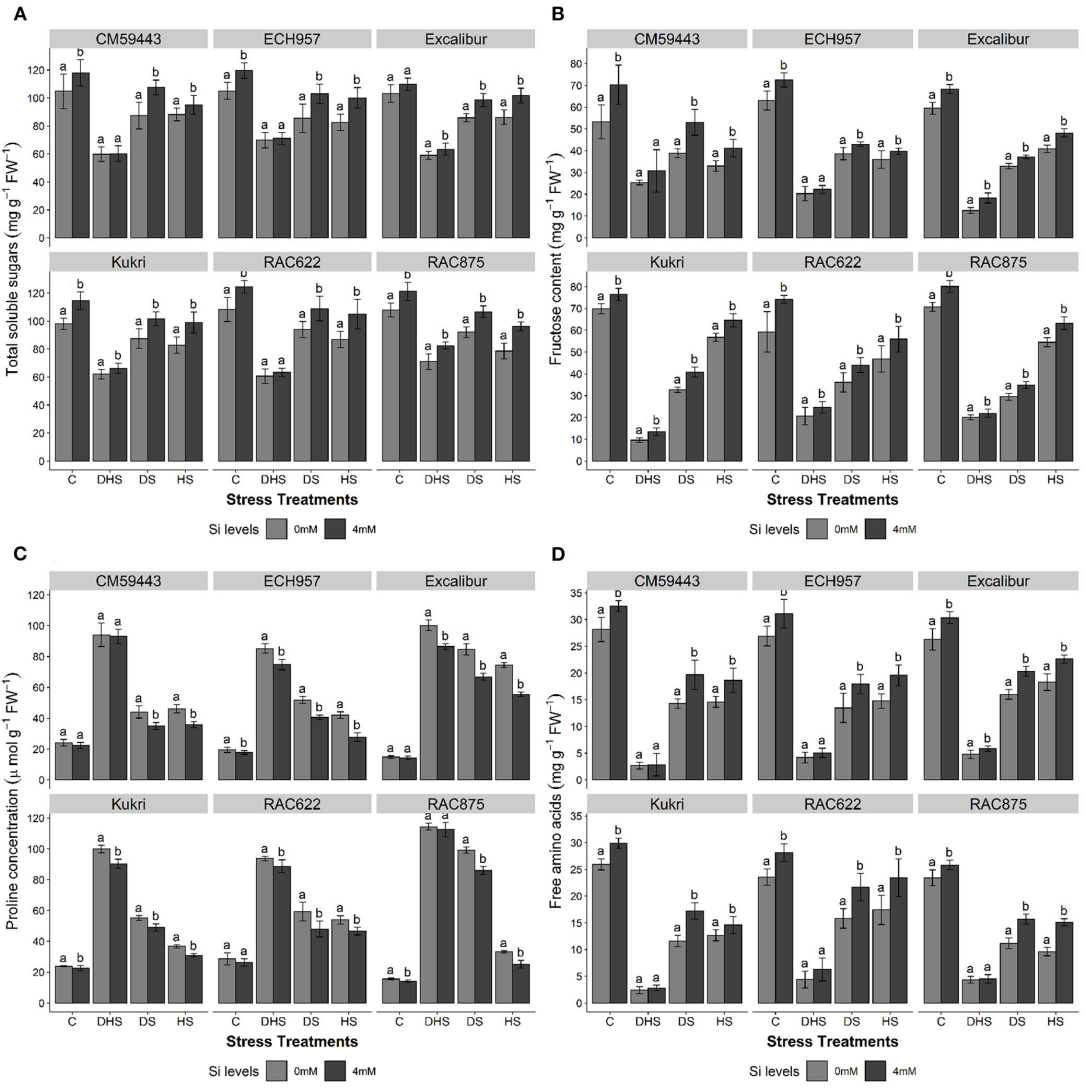
**(A–D)** Effect of silicon on **(A)** total soluble sugars (TSS) (mg g^−1^FW), **(B)** fructose content (FC) (mg g^−1^FW), **(C)** proline concentration (μmol g^−1^FW), and **(D)** free amino acids (AA) (mg g^−1^FW) of six contrasting wheat cultivars under terminal DS, HS, and DHS in a controlled glasshouse conditions. Mean values are presented with ± SE, represented with error bars. Different letters denote statistical significance within each cultivar at *p* < 0.05.

In the studied cultivars (Excalibur, RAC875, ECH957, RAC622, CM59443, and Kukri), Si treatment significantly (*p* ≤ 0.05) increased the concentrations of all estimated osmolytes in response to stress treatments (DS, HS, and DHS) except for the proline (Figures 4A–D). For TSS and FC, a considerable variation was observed across the cultivars under all stress treatments. Without Si treatment, the concentration of TSS was reduced by 11.9–22.9 % under DS, 18.9–37.4% under HS, and 50.4–78.6% under DHS compared to their respective controls (Figure 4A). Results indicated that Si treatment positively enhanced TSS concentration (*p* < 0.01). Under HSSi, the TSS concentration of tolerant cultivars increased significantly and ranged from 17.9 to 22.5%, compared with a 19.3 and 7.8% increase in Kukri and CM59443, respectively. Under DSSi, CM59443 had the highest increase (23.1%) in TSS compared with ECH957, which exhibited a maximum increase (20.5%) in TSS among the tolerant cultivars. Under DHSSi, RAC875 had the maximum increase in TSS (16.1%), followed by Excalibur (7.4%). Among the susceptible cultivars, the TSS of Kukri increases significantly (6.7%) under DHSSi. However, no significant effect of Si treatment on the TSS concentration was observed on RAC622, ECH957 (tolerant cultivars), and CM59443 (susceptible cultivar) under DHSSi.

The FC of all the cultivars increased with Si treatment under DS, HS, and DHS (*p* < 0.01). Under HSSi, the FC, compared with the non-treated controls, was markedly higher in the susceptible (CM59443, 36.6% and Kukri, 24.8%) and the tolerant cultivars (RAC875, 16.1%; RAC622, 19.2%; Excalibur, 17.9% and ECH957, 10.3%) (Figure 4B). Results showed that CM59443 had the maximum FC increase under DSSi followed by Kukri (24.8%), RAC622 (22.0%), RAC875 (18.2%), Excalibur (12.8%), and ECH957 (11.4%). Under HSSi, RAC875 and Kukri had similar FC (63.2 ± 3.0 and 64.6 ± 3.0 mg g^−1^ FW, respectively) compared with the 32% lower concentration in Excalibur (48.1 ± 1.9 mg g^−1^ FW). Under DHSSi, no significant effect of Si was observed in CM59443 and ECH957. The maximum increase under DSSi was observed in Excalibur (45.8%), followed by Kukri (39.7%), RAC622 (19.7%) and RAC875 (9.5%). This result showed that Si positively enhanced FC under DS, followed by HS and DHS.

Exposure to DS, HS, and DHS induced proline accumulation to a maximum level across the cultivars. The maximum proline concentration was observed under DHS across the cultivars. Under DS, proline concentration was found to be higher in the tolerant cultivars (86.0 ± 2.5 and 66.9 ± 2.2 μmol g^−1^ FW in RAC875 and Excalibur, respectively) when compared with the susceptible cultivars (49.0 ± 2.4 and 44.0 ± 3.9 μmol g^−1^ FW in Kukri and CM59443, respectively). However, Si treatment significantly reduced proline concentration across the cultivars (*p* < 0.001). Excalibur showed reduced proline concentration of 25.4, 21.0, and 13.8% under HS, DS, and DHS, respectively, followed by ECH957 under HS, DS, and DHS (34.0, 21.8, and 12.1% respectively). Among the susceptible cultivars, decreased proline concentrations of 16.0, 11.2, and 9.6% were observed in Kukri under HS, DHS, and DS, respectively, compared with their respective controls (Figure 3C). Cultivars RAC875 and CM59443 did not show any significant effect of Si treatment on proline concentration under DHS.

A significant increase (*p* < 0.001) in AA concentration was also observed with Si treatment across the cultivars (Figure 4D). Compared with the tolerant cultivars, a maximum decrease was observed in Kukri and CM59443 after the DH and HS. Under DSSi, the maximum increase in AA compared with respective control was observed in Kukri (48.3%), followed by RAC875 (40.5%) and CM59443 (37.7%). For HSSi, the tolerant cultivars performed better with Si treatment with a higher increase in AA than the susceptible cultivars. The RAC875 induced a significant increase of 57.0% in AA under HSSi, followed by 32.6% in RAC622. The Si-induced increase in AA was also recorded in CM59443 and Kukri by 27.5 and 15.1%, respectively. However, except for Excalibur, AA did not change significantly with Si treatment under DHS.

### Effects of Si on the antioxidant enzymes activities

The activities of enzymatic antioxidants, i.e., POX, CAT, and APX, were considerably increased under DS, HS, and DHS compared with the respective controls. However, results showed that Si treatment effectively further enhanced the activities of these antioxidants under all stress treatments (Table 1).

**Table 1:** Activity of enzymatic antioxidants of six contrasting wheat cultivars under drought stress (DS), heat stress (HS), and drought-heatcombined stress (DHS) in controlled glasshouse conditions.

	**Traits**	**Treatment**	**C**		**DS**		**HS**		**DHS**	
		**Cultivars**	**-Si**	**+Si**	**-Si**	**+Si**	**-Si**	**+Si**	**-Si**	**+Si**
Enzymatic antioxidants	POX	RAC875	3.3 ± 0.8a	4.2 ± 0.5a	6.3 ± 0.6a	8.2 ± 05b	8.3 ± 0.6a	9.8 ± 1.0b	10.7 ± 1.1a	11.1 ± 1.1a
		Kukri	2.4 ± 0.2a	3.2 ± 0.4b	6.1 ± 1.16a	8.0 ± 0.2b	8.5 ± 0.4a	9.9 ± 0.7b	10.9 ± 0.4a	11.5 ± 1.2a
		Excalibur	2.2 ± 0.5a	2.9 ± 0.3b	6.0 ± 0.5a	7.8 ± 0.7b	7.7 ± 0.7a	9.4 ± 0.5b	10.2 ± 0.7a	11.0 ± 1.1b
		ECH957	4.1 ± 0.3a	4.6 ± 0.4a	6.7 ± 0.4a	7.8 ± 0.1b	6.9 ± 0.2a	8.0 ± 0.2b	10.1 ± 0.3a	11.0 ± 0.6b
		RAC622	4.4 ± 0.7a	5.1 ± 0.7a	7.3 ± 0.5a	8.7 ± 0.7b	8.7 ± 0.3a	10.3 ± 0.5b	11.4 ± 0.6a	12.0 ± 0.7a
		CM59443	4.0 ± 0.6a	4.6 ± 0.3a	6.8 ± 0.5a	7.9 ± 0.6b	8.6 ± 0.3a	9.4 ± 0.6b	11.6 ± 0.3a	11.9 ± 0.6a
										
	CAT	RAC875	5.5 ± 0.9a	6.9 ± 0.7b	17.4 ± 1.3a	22.3 ± 2.7b	15.0 ± 2.1a	20.6 ± 1.3b	32.4 ± 3.5a	36.7 ± 1.6b
		Kukri	8.2 ± 1.3a	9.3 ± 0.7a	18.1 ± 1.8a	25.3 ± 1.9b	17.1 ± 1.6a	21.5 ± 1.5b	34.7 ± 1.5a	39.9 ± 2.8b
		Excalibur	9.4 ± 0.6a	12.0 ± 1.0b	21.5 ± 0.1a	26.8 ± 2.1b	18.8 ± 1.1a	24.4 ± 1.5b	34.9 ± 1.4a	38.5 ± 2.1b
		ECH957	8.4 ± 1.1a	9.7 ± 1.8b	18.7 ± 0.1a	21.2 ± 0.9b	20.3 ± 1.4a	21.5 ± 1.1b	28.6 ± 1.3a	31.7 ± 2.4b
		RAC622	10.2 ± 1.1a	10.5 ± 1.8a	21.1 ± 0.9a	22.8 ± 0.9b	21.6 ± 1.4a	23.9 ± 1.2b	30.5 ± 1.9a	33.0 ± 2.5b
		CM59443	10.4 ± 0.7a	11.4 ± 0.4b	23.3 ± 1.4a	25.2 ± 1.4b	19.7 ± 1.0a	21.1 ± 0.1b	32.4 ± 2.1a	33.2 ± 1.2a
										
	APX	RAC875	12.9 ± 2.2a	15.7 ± 1.4b	45.2 ± 2.1a	51.9 ± 5.0b	53.3 ± 1.3a	59.5 ± 3.1b	65.7 ± 3.0a	71.9 ± 1.7b
		Kukri	7.1 ± 1.4a	8.6 ± 2.2a	39.0 ± 4.2a	44.8 ± 3.1b	48.1 ± 1.7a	51.4 ± 2.5b	57.6 ± 1.3a	59.5 ± 1.7b
		Excalibur	11.9 ± 2.7a	14.3 ± 0.8a	45.7 ± 3.0a	49.0 ± 2.4b	48.1 ± 0.5a	52.4 ± 1.3b	59.0 ± 1.7a	61.9 ± 2.1b
		ECH957	11.9 ± 0.5a	12.9 ± 0.8a	44.8 ± 1.7a	46.7 ± 1.3b	42.4 ± 1.3a	45.2 ± 0.5b	51.9 ± 1.3a	55.2 ± 1.3b
		RAC622	21.4 ± 0.8a	22.9 ± 0.8a	39.5 ± 1.7a	41.9 ± 1.0b	43.3 ± 1.7a	52.4 ± 1.3b	50.5 ± 1.7a	52.9 ± 0.8b
		CM59443	17.1 ± 0.8a	19.5 ± 1.3b	41.9 ± 1.7a	45.2 ± 0.8b	45.7 ± 0.8a	48.6 ± 0.8b	57.1 ± 0.8a	59.0 ± 2.1a

POX, Peroxidase; CAT, catalase; APX, ascorbate peroxidase. Each value represents the mean ± SE. Alphabets denote statistical significance at p < 0.05 within cultivars and treatment effect.

Silicon treatment significantly enhanced the POX activity under all stress treatments (*p* < 0.01). Under DSSi, compared to the DS (no Si), the POX activity was significantly elevated across all the cultivars by 15.4–30.3%. The maximum Si-induced increase in the POX activity under DSSi was observed in Kukri (30.3%), followed by RAC875 (30.2%), compared to CM59443 with the least increase in POX activity with Si (15.4%). Whereas, under HSSi, the increased percentage ranged from 9.3 to 22.1% among the cultivars. Under HSSi, the maximum increase in the POX was observed in Excalibur (22.1%), followed by RAC622 (19.1%). Kukri and CM59443, susceptible cultivars, had a 16.5 and 9.3% increase in Si-induced POX activity under HSSi. Under DHSSi, ECH957 and Excalibur exhibited the most significant increase in the POX activity (8.9 and 7.8%, respectively) compared with Kukri and CM59443 with the slightest increase in POX activity (5.5 and 2.2%, respectively).

Similarly, Si treatment exerted a significant (*p* < 0.01) positive effect in enhancing the CAT activity across the cultivars. With Si treatment under DS (DSSi), the CAT activity was significanlty enhanced by 28.5, 24.7, 13.6, and 8.2% in RAC875, Excalibur, ECH957, and RAC622 (tolerant cultivars), respectively, compared with a 39.8% increase in Kukri and an 8.3% in CM59443 (susceptible cultivars). Under HSSi, the CAT activity was also accelerated by 36.9, 29.9, 10.3, and 6.0% in RAC875, Excalibur, RAC622, and ECH957 (tolerant cultivars), respectively, compared with a 25.9% increase in Kukri and a 7.2% increase in CM59443 (susceptible cultivars). Although, compared with DSSi and HSSi, the CAT activity was highest under DHSSi, but the increased percentage with Si treatment was minimum across the tolerant cultivars (RAC875 = 13.4%, Excalibur = 10.6%, ECH957 = 8.9%, RAC622 = 8.2%), compared to susceptible cultivar, Kukri (15.2%) (Table 1).

For APX, a significant (*p* < 0.001) increase with Si was observed across all the cultivars under DS, HS, and DHS treatments. Under DSSi, the increase in APX activity ranged between 4.3 and 14.7% across the cultivars and the highest increase was observed in RAC875 (14.7%) and Kukri (14.6%). Similarly, under HSSi, the increase in the APX activity ranged between 6.3 and 20.9%, and the highest APX activity was observed in RAC622 (20.9%), followed by RAC875 (11.6%). The APX activity under HSSi was significantly enhanced in susceptible cultivars as well, and it was 6.3% and 6.9% in CM59443 and Kukri, respectively. Under DHSSi, the highest APX activity was recorded in RAC875 (9.4%), followed by ECH957 (6.4%) as compared with a 3.3% increase in each of the susceptible cultivars (Kukri and CM59443) (Table 1).

### GY and yield components

**Figure 5:**
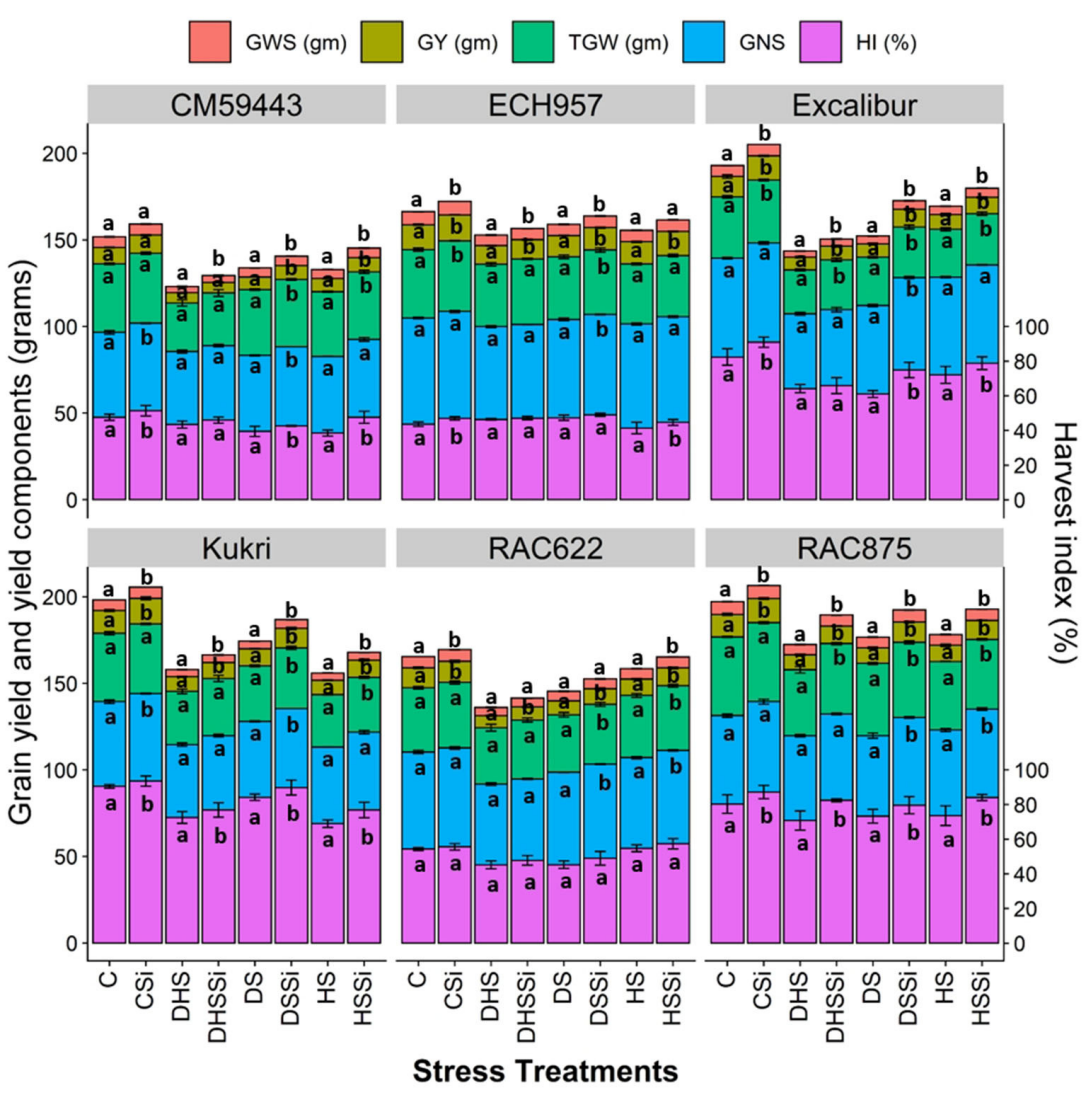
Effect of silicon on the mean values of grain yield (GY) and yield components of six contrasting wheat cultivars under DS, HS, and DHS in a controlled glasshouse condition. Each value represents the mean ± SE, represented with error bars. Different letters indicate significant differences within the cultivars at *p* < 0.05. Abbreviations are grain weight per spike (GWS, grams; presented as GWS ×10^∧^0.5), grain yield per pot (GY, grams), 1,000 grains weight (TGW, grams), number of grains per spike (GNS), and harvest index (HI, %).

Under C, all cultivars performed their best yield potential and were found to have comparable GY (Figure 5). In contrast, experimental stress treatments resulted in a significant reduction in GY among the cultivars. Up to 66 % GY reduction was observed under DHS across all the cultivars and the lowest yield was recorded in CM59443 (5.9 ± 0.2 gm pot^−1^). The reduction in GY under HS was the highest in Kukri (58.8%), followed by RAC875 (39.1%) and Excalibur (38.8%) compared with the C. Similarly, DS treatment resulted in significant GY losses in the susceptible cultivars CM59443 and Kukri, and they yielded 56 and 10.1% less GY than the average GY of tolerant cultivars. However, the Si treatment compared with controls resulted in significantly (*p* < 0.001) higher GY across the cultivars under all the stress treatments. Cultivars RAC875 and Excalibur produced 3 folds (~30%) extra GYunder DSSi than Kukri and CM59443 (~10%). Under HSSi, the GY was improved by 12.2% when averaged across the cultivars. When compared among cultivars, Kukri yielded 20.2% more grains than the untreated C and 16.6% extra grains than RAC875 under HSSi. Under DHSSi, a maximum increase was observed in RAC875 (16.5%), followed by RAC622 (9.7%) and Kukri (8.11%). Overall, the tolerant cultivars produced a higher GY (40.7%) than susceptible cultivars (31.9%) under all stress treatments with Si effects.

Different stress treatments significantly reduced the GY of wheat cultivars and hence the HI (Figure 5). The reduction in the HI of the cultivars under DS, HS, and DHS ranged between 6.9 and 25.9%, 8.3–31.1%, and 13.4–28.4 %, respectively, compared with the C treatment. With Si treatment, a significant increase in the HI was observed across all the stress treatments. Excalibur had the highest HI increase (18.4%) under DSSi and CM59443 had the highest growth in HI (23.6%) under HSSi compared with the C treatment. On average, susceptible cultivars produced 16.4% more HI with Si treatment across the stress treatments than the tolerant cultivars.

Like GY, TGW reduced significantly with DS, HS, and DHS. A significant increase (*p* < 0.01) in TGW was observed with Si across the stress treatments. Silicon treatment induced a 2.6–9.3% increase in TGW under DS, 1.4–7.3% increase under HS, and 4.4–13.8% increase under DHS when averaged across the cultivars. The highest averaged Si-induced increase in TGW across the stress treatments was observed in Excalibur (8.6%), followed by Kukri (6.9%) and CM59443 (4.9%). When compared within DSSi, Kukri increased TGW twice (9.4%) as compared with RAC875 (4.1%) and Excalibur (4.8%) and almost four times when compared with ECH957 (2.6%). Similarly, under HSSi, Excalibur had the highest increase (7.3%) in TGW among the tolerant cultivars, compared with Kukri with 4.5% increase among the susceptible cultivars. Similarly, Excalibur obtained the maximum TGW (13.8%), followed by CM59443 (8.1%) under DHSSi.

For grains per spike, stress treatment caused a significant decrease across all the cultivars. The highest average GNS decrease across the cultivars was observed in DHS (17.3%), followed by DS (9.7%) and HS (5.9%). Results showed that Si treatment significantly (*p* < 0.05) increased the GNS under DS, HS, and DHS across the cultivars. Excalibur, CM59443, and Kukri had increased GNS with almost the same number (4.5%) compared with a 2-fold increase in RAC875 (8.8%) under DS. For HSSi, RAC875 and RAC622 had increased GNS by 3.5 %. However, the no-significant effect of Si on the GNS was observed under DHSSi across the cultivars.

Similarly, GWS was severely impacted by stress treatments across the cultivars. Compared with C, the GWS was severely reduced by up to 80, 41.8, and 44.3% under DHS, DS, and HS, respectively. Results showed that Si treatment significantly increased the GWS by up to 5.0–13.8% in DS, 2.4–7.8% in HS, and 5.0–13.7% in DHS. Overall, the maximum Si-induced increase in GWS was observed in Excalibur (10.4%), followed by the susceptible cultivars, Kukri (9.8%) and CM59443 (7.2%).

## Discussion

### RWC, chlorophyll fluorescence, and chlorophyll contents

The RWC is an important physiological attribute for assessing cell and tissue dehydration during DS and HS. Higher RWC is a result of higher osmotic regulations and confers normal metabolic activity and other adaptations by plants (Erice et al., 2010). In the current study, severe RWC reduction was observed under DHS, followed by DS and HS across the studied cultivars, suggesting that combined stress is more detrimental for plants irrespective of their tolerance level. Our study reported that Si treatment helped maintain leaf osmotic potential (except in susceptible cultivars under DHS) and RWC at a higher level due to the net accumulation of various solutes in the symplast (Figures 4A–D). However, Alzahrani et al. (2018) suggested that Si treatment lowered the ion leakage and improved the cell membrane integrity under stressful environments. To ensure maximum productivity under DS and HS, wheat cultivars with higher RWC might have maintained protoplast hydration for long (Biju et al., 2018; Sattar et al., 2020). Higher RWC also links with higher photosynthetic activity, stomatal conductance, and leaf transpiration, leading to sustained plant growth and GY.

Cultivars with higher chlorophyll fluorescence (F_v_/F_m_) and chlorophyll contents (SPAD value), after stress treatments have been shown to stay green for extended periods (Murchie and Lawson, 2013). In this study, Kukri (a susceptible cultivar) senesced earlier due to stress-induced pale green leaves and lower chlorophyll contents than RAC875 and Excalibur, which maintained dark green leaves. The result showed that the pre-sowing treatment of Si alleviated the inhibition of photosynthetic apparatus caused by DS, HS, and DHS through improved photochemical efficiency of the PSII photosystem. The Si treatment decreased the oxidative stress during terminal DS and HS and was accompanied by higher F_v_/F_m_ (Figure 2A) (Izanloo et al., 2008). In contrast, high leaf senescence and leaf abscission are significantly accelerated under DHS (Wang et al., 2011). Although Si treatment helped alleviate F_v_/F_m_ level under DHS but to a non-significant level, it shows DHS that affects plants, leading to reduced and/or ceasing of photosynthesis due to the early onset of senescence, which leads to yield losses in grain crops.

### Computational water stress indices

In the current study, thermal imaging analysis has successfully been used as an integrative approach to accurately estimate T_c_, CTD, and CWSI under DS, HS, and DHS. Previously, T_c_ has been used by many researchers as a reliable measure of the overall plant water status and an important selection criterion for the DS environment (Fuentes et al., 2012; Costa et al., 2013; Biju et al., 2018). In a well-watered condition, water normally transpires through open stomata to maintain T_c_ below T_a_. However, results showed that DS, HS, and DHS directly affected the T_c_ of all the cultivars, resulting in increased transpiration and later higher T_c_, especially in susceptible wheat cultivars. Similar or higher T_c_, compared with T_a_ under stressful conditions (DS, HS, and DHS), is due to the closing of stomata as a preventive measure to maintain leaf turgor pressure. Results showed that Si treatment led to a significant reduction (*p* < 0.01, up to 4.4%) in T_c_ across the cultivars under all stress treatments. These findings confirm that added Si helps plants maintain adequate water status by keeping low T_c_ compared to T_a_ for optimum photosynthetic activity under DS, HS, and combined DHS. Under DHS, Kukri exhibited a significantly lowered T_c_ with Si treatment, which showed that Si has the potential to alleviate the effects of combined DHS and HS even in susceptible wheat cultivars (Figure 3A).

Canopy temperature depression has been categorized as an important trait for the overall plant water stress status and is directly correlated with the transpirational status of the crops. It is the key plant attribute for comparing/assessing genotypic response to environmental stresses, i.e., DS or HS (Balota et al., 2007), and positively correlated with GY under stressful environments. The current study showed that CTD decreased with the severity of the stress, and the maximum severity was observed in susceptible cultivars under DHS (Figure 3B), which is understandable as combined stress adds to the degree of complexity compared to individual stresses. The differences observed in CTD values for the tolerant and susceptible cultivars are possibly due to canopy development differences due to their different genetic constitutions among cultivars and cultivars × environment (G × E) interaction in a controlled environment. The Si treatment improved the CTD by maintaining cell turgor through osmotic adjustments in the plant cells. Our results suggest that individual and combined DHS during the early grain-filling stage can be critical for employing CTD in explaining the GY variation and its related components with and without Si treatment. The grain-filling stage is more vulnerable to DS and HS, mainly affecting grains per spike and thousand-grain weight. However, cooler T_c_ and higher CTD could improve the photosynthesis during grain filling and, consequently, final GY under stressful environmental conditions.

**Figure 6:**
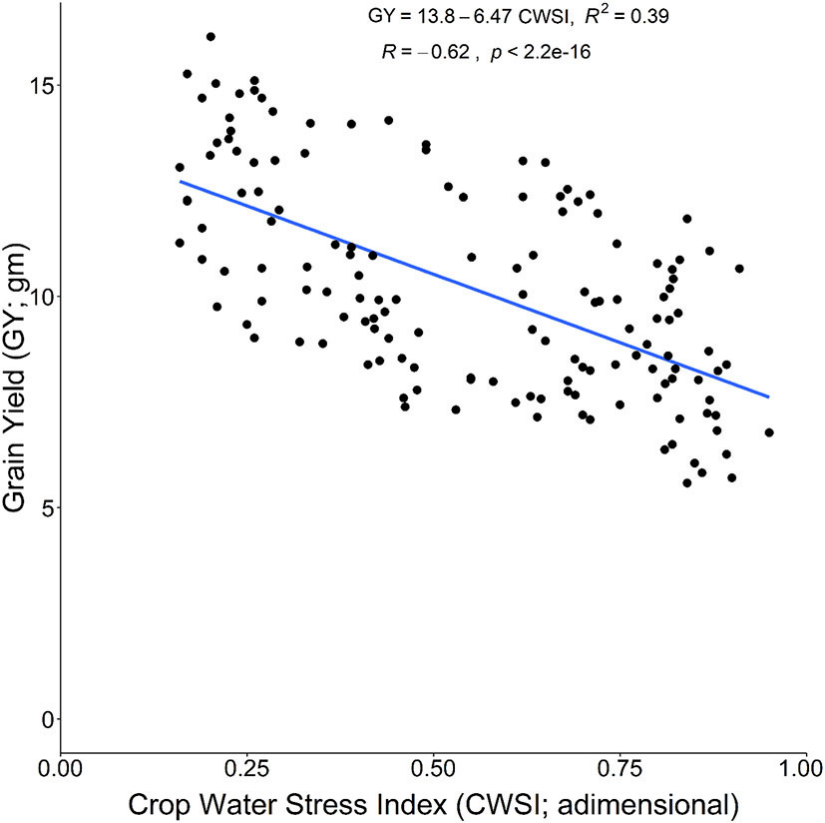
Relationship between GY across the cultivars as the dependent variable with crop water stress index as an independent variable under DS, HS, and DHS conditions.

The CWSI is an important indicator for plant water status based on T_c_. The CWSI fluctuated with the various stress treatments of DS, HS, and DHS, and previously, it was successfully used to quantify the plant water status under DS (Biju et al., 2018). The CWSI showed a significant increase in the current study under DS, HS, and DHS irrespective of the tolerant and susceptible cultivars. However, the Si treatment positively reduced the CWSI across the stress treatments (Figure 3C). Compared to the tolerant cultivars, susceptible cultivars also showed a significant decrease in CWSI with Si treatment under DS, HS, and DHS. The reduction in CWSI might be linked with Si-induced improved osmotic adjustments, and increased plasma membrane aquaporins function for higher root water uptake under abiotic stress environments (Shi et al., 2016). Also, CWSI has a strong negative correlation with the GY under DS and HS. Results showed that GY increased significantly with the decrease in CWSI of the tolerant and susceptible wheat cultivars. The relationship between GY (as a dependent variable) and CWSI (as an independent variable) is presented in Figure 6. It indicates GY declined with increasing CWSI (*R*^2^ = 0.39). This result agrees with the previous findings in wheat (Alghory and Yazar, 2019). It also shows a direct linear relationship between T_c_, which means CWSI increases with the increase in T_c_.

### Osmolytes

The most common stress tolerance mechanisms in plants against DS, HS, and DHS are the excessive production of various osmolytes (TSS, FCs, proline, and free AA), the first adaptive mechanism in response to adverse environmental conditions. The synthesis of these compatible solutes is widely recognized as essential for plant adaptation in cellular dehydration and stabilizes various enzymatic antioxidants to contribute to abiotic stress tolerance (Biju et al., 2017; Sattar et al., 2020).

In the current study, DS, HS, and DHS induced a significant adverse effect on producing different osmolytes/solutes in the tolerant and susceptible cultivars. Various osmotic adjustments under DS and HS are innate behavior of plants that help them maintain water balance across the cells by synthesizing various osmolytes. The current study results showed that Si treatment significantly increased the TSS and FCs in tolerant and susceptible wheat cultivars under DS, HS, and to some extent in DHS (Figures 4A,B). Added Si under DS and HS might enhance the anabolism of organic solutes to maintain cell turgor and protect the cell structures and functions from dehydrative damage (Pei et al., 2010; Alzahrani et al., 2018). Also, Si-induced increase in cell water resulted in minimum cellular dehydration (Pei et al., 2010) and, therefore, less oxidative stress due to low ROS levels, which directly enhanced the tolerance levels of susceptible and tolerant wheat cultivars under DS, HS, and to some extent in DHS. Additionally, Si-induced accumulation of higher organic solutes might be available for quick breakdown at the end of stress (DS, HS, and DHS) as a source of energy to repair damaged cells and tissues. From the current study results, it can be concluded that Si-induced higher accumulation of various osmolytes might also be linked with increased activity of different antioxidants for the scavenging of overproduced ROS to enhance HS and DS tolerance in the susceptible wheat cultivars, as well as the tolerant cultivars. Therefore, based on the results, we suggest that the synergistic association of various organic solutes with antioxidants enhances DS and HS tolerance in bread wheat (Abid et al., 2018).

In the present study, under DS, HS, and DHS, both tolerant and susceptible cultivars had increased biosynthesis and accumulation of proline. Under stressful conditions, increased proline concentration is linked with stress tolerance and helps maintain the osmotic potential for alleviating stress effects across the cultivars. Added Si led to a significant adjustment to proline biosynthesis both in the tolerant and the susceptible wheat cultivars, particularly under DS and HS, which might show that stress relief responses due to alleviation of various cellular damages, including oxidative burst and peroxidative damage to the cell membrane (Figure 4C). Consistent with the results presented here, several other studies have also reported that Si application reduces the proline accumulation on multiple crops like wheat (Pei et al., 2010), rice (Mauad et al., 2016), lentil (Biju et al., 2017), and sorghum (Yin et al., 2013). Results showed that Si might have a definite controlling effect on proline biosynthesis, as reported by Vivet et al. (2000). The Si-induced breakdown of proline due to the controlling effect may support the generation of ATP to repair stress-induced damages in the cytosol for substantial cytoplasmic osmotic adjustments of treated wheat cultivars (Ashraf and Foolad, 2007). Accumulation of higher proline concentration under stressful conditions has been correlated in tolerant cultivars more than in susceptible cultivars, as reported by Ashraf and Foolad (2007) but results from the current study showed that this relationship might not be universal. For example, proline accumulation under HS was higher in Kukri than in the tolerant cultivar, RAC875. Similarly, CM59443 accumulated more proline than ECH957 under DHS (Figure 4C).

Free AA concentrations increased across the tolerant and susceptible wheat cultivars with Si treatment under DS and HS (Figure 4D). Results showed that DHS significantly impacted the free AA concentration, and no significant increase in AA was observed with Si treatment across the wheat cultivars (except Excalibur). The percent increase in free AA in tolerant and susceptible wheat cultivars might be associated with the enhanced production of various antioxidants in response to DS and HS. The ROS scavenging minimizes AA oxidation, which could help maintain the integrity of protein structure under DS and HS as reported by Ashraf and Foolad (2007). Previously, Cvikrová et al. (2013) reported that enhanced free AA concentration partially converted to proline to relieve DS and HS effects. Si-induced increase in free AA and TSS helps plants to make structural and biochemical adjustments to cope with stress environments (Sallam et al., 2019).

### Antioxidant enzymes

With normal cellular metabolisms, plants continuously produce various ROS. The exposure of plants to individual and combined DS and HS during their life cycle disturbs the quenching activity of different antioxidants, especially in susceptible cultivars, leading to excessive production of several ROS (Alzahrani et al., 2018). Plants with higher antioxidant levels, both in constitutive or induced form, have demonstrated maximum tolerance against oxidative stress damages to cells and organelles.

In the current study, APX, POX, and CAT activities were triggered with the onset of DS, HS, and DHS. However, the increase in the activities of these antioxidants was higher with Si treatment than with control across the cultivars (Table 1). Si-induced increased APX activity across the cultivars (tolerant and susceptible) suggests Si treatment might dismutase O${}_{2}^{-}$ to H_2_O_2_ in the mitochondria, chloroplast, and cytoplasm to prevent cellular damage under DS, HS, and DHS. Accumulation of H_2_O_2_ that results from APX canalization reaction is further detoxified by the POX-mediated ascorbate-glutathione cycle. Silicon-induced higher CAT activity eliminates H_2_O_2_ by breaking it down into oxygen and water molecules. Other research findings have also shown that higher POX activity is correlated with increased stress tolerance to various oxidative stresses (Alzahrani et al., 2018; Etesami and Jeong, 2018). DS- and HS-induced OH^−^, coupled with H_2_O_2_ can react with macromolecules without discrimination, facilitating cellular damage across the cultivars. The results showed that Si-induced integrating activities of APX, CAT, and POX under DS, HS, and DHS could inhibit or reduce OH^−^ formation among the tolerant and susceptible wheat cultivars; similar results have been reported by Pei et al. (2010) and Biju et al. (2017). The increased APX, POX, and CAT activities with Si treatment prevented the studied wheat cultivars from cellular damage with improved tolerance to DS, HS, and DHS.

### GY and yield components

Abiotic stresses, particularly combined DHS, have detrimental impacts on GY and yield-related components (Reynolds et al., 2005). Genotypic efficiency for stress tolerance is usually quantified by GY and its related components under a stressful environment and mainly depends on the degree and severity of the stress treatment. In the current experiment, genotypes × environmental interaction was highly variable, showing that all cultivars responded differently to control and stress treatments (DS, HS, and DHS). A significant difference was observed among tolerant and susceptible cultivars in their response to DS, HS, and DHS. The differential sensitivity of the cultivars for various yield components, such as HI, thousand grains weight, GNS, and grain weight per spike to DS, HS, and DHS was indicative of their varied response (Figure 5). With added Si, while encountering stress, susceptible cultivars also performed better and produced higher GY and yield components (TGW and GWS). Compared to the control treatment, this recovery might be due to enhanced photosynthetic assimilates linked with Si-induced higher photosynthesis and soluble carbohydrates under DS and HS (Figure 4A).

## Conclusion

Terminal DS and HS frequently occur in most of the wheat belt, which reduces grain weight, grain number, and ultimately, final GY. The current study results suggested that Si inclusion as a nutritional element is beneficial under normal and adverse environmental conditions. Si treatment has maintained various physiological and biochemical attributes by regulating the leaf water potential under DS, HS, and DHS both in the tolerant and susceptible wheat cultivars. In addition, our findings also validated that tolerance to DS and HS in susceptible and tolerant wheat cultivars with pre-sowing Si treatment was linked with the enhanced antioxidant defense activities and alleviation of oxidative stress damages. Results showed that Si-induced higher percent improvement in morpho-physiological and biochemical traits was observed under DS, compared with HS and DHS. Overall, RAC875 among the tolerant cultivars and Kukri among the susceptible cultivars performed better with Si treatment across the stress treatments. However, a better understanding of individual and combined stress and the response of different cultivars with and without Si under field conditions will assist in validating controlled study results.

## Data availability statement

The raw data supporting the conclusions of this article will be made available by the authors, without undue reservation.

## Author contributions

Conceptualization and writing—review and editing: WA, DG, SF, and GB. Methodology: WA and DG. Supervision and funding acquisition: DG. Investigation, data curation, statistical analysis, and writing—original draft preparation: WA. Resources and equipment: DG, SF, and GB. Software: WA and SF. All authors have read and approved the final version of the manuscript.

## Conflict of interest

The authors declare that the research was conducted in the absence of any commercial or financial relationships that could be construed as a potential conflict of interest.

## Publisher's note

All claims expressed in this article are solely those of the authors and do not necessarily represent those of their affiliated organizations, or those of the publisher, the editors and the reviewers. Any product that may be evaluated in this article, or claim that may be made by its manufacturer, is not guaranteed or endorsed by the publisher.
